# Nationwide Outcomes of Heart Transplantation for Postpartum Cardiomyopathy

**DOI:** 10.31083/RCM25831

**Published:** 2025-01-09

**Authors:** Ilias P. Doulamis, Aspasia Tzani, Ahmet Kilic, Toshiki Kuno, Alexandros Briasoulis

**Affiliations:** ^1^Department of Surgery, Lahey Clinic, Burlington, MA 01805, USA; ^2^Divison of Cardiothoracic Surgery, Department of Surgery, Johns Hopkins University School of Medicine, Baltimore, MD 21287, USA; ^3^Department of Cardiovascular Medicine, Brigham and Women’s Hospital Heart and Vascular Center, Harvard Medical School, Boston, MA 02115, USA; ^4^Division of Cardiology, Montefiore Medical Center, Albert Einstein College of Medicine, Bronx, NY 10467, USA; ^5^Division of Cardiovascular Medicine, Section of Heart Failure and Transplantation, University of Iowa, Iowa City, IA 52242, USA

**Keywords:** UNOS, heart transplantation, postpartum cardiomyopathy

## Abstract

**Background:**

Postpartum cardiomyopathy is defined as an incident of acute heart failure in the postpartum period in the absence of any other cause. Up to 10% of postpartum cardiomyopathy may need to undergo heart transplantation later in life. This study aimed to provide a present-day perspective on all-cause mortality and transplant-related complications after heart transplantation for postpartum cardiomyopathy.

**Methods:**

A retrospective analysis of the United Network for Organ Sharing (UNOS) registry was performed for adult patients undergoing heart transplants (01/2001–01/2023) for postpartum cardiomyopathy.

**Results:**

A total of 677 patients were identified, with a mean age of 35 years. The mean body mass index (BMI) was 27.2 kg/m^2^; the most common comorbidity was type 2 diabetes (T2D) (n = 589; 87%). Older age was associated with lower overall mortality (hazard ratio (HR): 0.97; 95% CI: 0.95, 0.98; *p* < 0.01), while diabetes (HR: 1.01; 95% CI: 1.01, 1.01; *p* < 0.01), dialysis (HR: 1.01; 95% CI: 1.01, 1.01; *p* < 0.01), days on Status 1 on the UNOS registry (HR: 1.06; 95% CI: 1.03, 10.9; *p* < 0.01), creatinine (HR: 1.29; 95% CI: 1.02, 1.64; *p* = 0.034), and length of stay (HR: 1.01; 95% CI: 1.01, 1.02; *p* = 0.02) were associated with a higher risk of overall mortality. Moreover, 30-day mortality was 2.8%, and 1-year mortality was 11.1%. The era effect was prominent in cases of 1-year mortality (odds ratio (OR): 0.95; 95% CI: 0.91, 0.99, *p* = 0.006).

**Conclusions:**

Our results suggest that younger age, diabetes, pretransplant dialysis, days on Status 1, and creatinine are associated with higher mortality, while an era effect was observed for 1-year mortality after heart transplantation (HTx) in patients with postpartum cardiomyopathy.

## 1. Introduction

Postpartum cardiomyopathy is defined as an incident of acute heart failure (HF) associated with systolic dysfunction (ejection fraction <45%, with or without ventricular dilatation) towards the end of pregnancy or in the postpartum period without any other cause or underlying cardiac disease [1]. Full recovery is expected in half of patients; however, variable outcomes have been reported for the other half. Current data suggest an approximate risk of 5% for mortality, heart transplantation (HTx), or need for left ventricular assist device (LVAD) implantation one year after diagnosis. Further literature reports that up to 10% of this population may need to undergo HTx later in life [2, 3]. This study aimed to provide a present-day perspective on all-cause mortality and transplant-related complications after HTx for postpartum cardiomyopathy.

## 2. Patients and Methods

### 2.1 Study Design

This is a retrospective United Network for Organ Sharing (UNOS) registry analysis. Deidentified patient-level variables for postpartum cardiomyopathy were collected from all adult patients (≥18 years of age) who underwent single-organ heart transplants between January 1st, 2001, and January 30th, 2023. Non-adult patients or patients who underwent other organ transplants simultaneously were excluded to minimize confounders. A total of 677 patients were included in our study. Baseline recipient characteristics included age, body mass index (BMI), ethnicity, comorbidities, sensitization status and pretransplant hemodynamics, recipient status, new allocation system implementation, mechanical assist device, and creatinine. Donor characteristics included age, sex, ethnicity, diabetes, creatinine, left ventricular ejection fraction (LVEF), distance, and ischemic time of the graft.

### 2.2 Outcome Measures

The primary outcomes of the study were 30-day and all-cause mortalities. Secondary outcomes included the need for pacemaker placement, dialysis, stroke, length of stay, and treatment for rejection within the first year after transplant.

### 2.3 Statistical Analysis

All continuous variables are expressed as the mean ± standard error of the mean, while categorical variables are expressed as frequencies and percentages. The normality of the distribution was assessed using the Shapiro–Wilk test. Meanwhile, Kaplan–Meier curves were created for overall survival. Univariable Cox regression was used for all-cause mortality, while univariable logistic regression was used for the rest of the outcomes. Univariable logistic regression analysis was utilized for binary outcomes. Multivariable analysis of the statistically significant variables in the univariable models was deferred due to the small number of significant predictors. Analyses were performed using Stata 17.0 (StataCorp. 2017, College Station, TX, USA). All tests were two-sided, and *p* < 0.05 was considered statistically significant.

## 3. Results

**Fig. 1.**
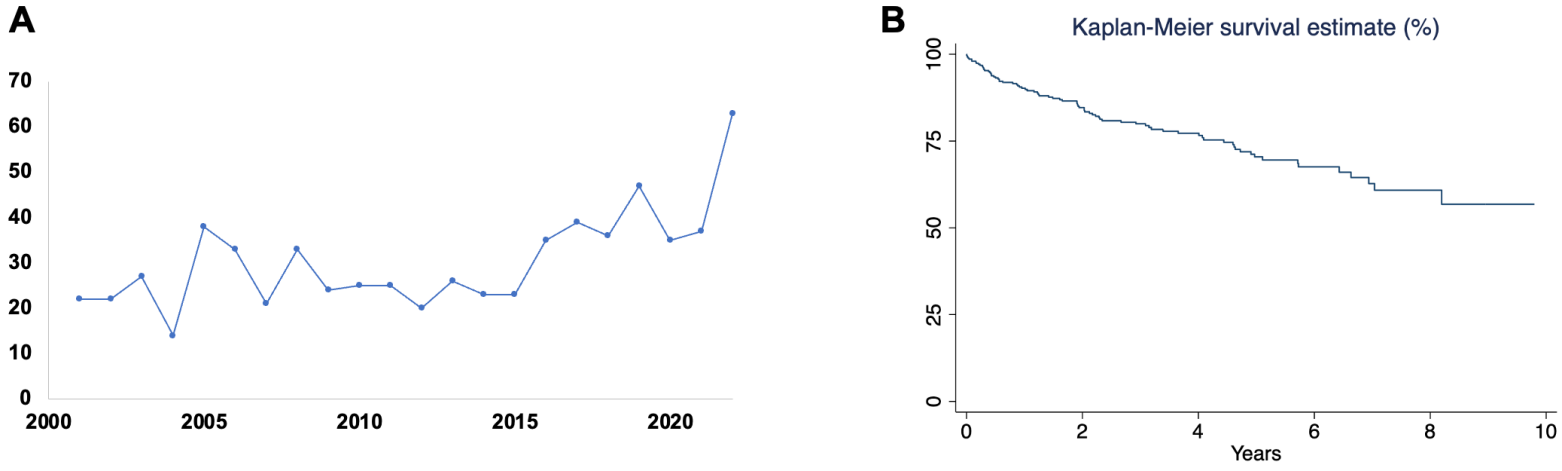
**Incidence and survival outcomes of postpartum cardiomyopathy requiring heart transplantation**. (A) The dot plot depicts the number of patients undergoing heart transplants for postpartum cardiomyopathy over time; (B) Kaplan–Meier curve shows the survival rate of patients undergoing heart transplants for postpartum cardiomyopathy over 10 years.

A total of 677 patients were identified. The average age was 35 years, and most of the women were black (n = 345; 51%). The mean BMI was 27.2 kg/m^2^; the most common comorbidity was type 2 diabetes (T2D) (n = 589; 87%). More than half of the patients had an implantable cardiac defibrillator (ICD) (n = 371; 55%), while approximately one out of ten patients had either a left ventricular assist device (LVAD) (n = 72; 11%) or a total artificial heart (TAH) (n = 68; 11%). Intra-aortic balloon pump (IABP) was used in 67 (10%) patients when wait-listed, while this number increased to 95 (14%) at the time of transplantation. A total of 135 patients (20%) had a previous cardiac surgery operation, and 95 (14%) were intravenous drug users (IVDUs). Average creatinine, calculated panel reactive antibody (CPRA), pulmonary capillary wedge pressure (PCWP), and mean pulmonary arterial pressure (mPAP) were 1.08 ± 0.02 mg/dL, 29.2 ± 2.3, 20.1 ± 0.4, and 27.9 ± 0.4 mmHg, respectively (Table 1). In the study population, 477 (70%) patients received a heart transplant using the old UNOS allocation system, while 200 (30%) patients after implementing the new UNOS donor allocation system in October 2018. From 2001 to 2022, there was an increasing trend in the number of patients with postpartum cardiomyopathy undergoing a heart transplant (r = 0.6; *p* < 0.01) (Fig. 1A).

**Table 1.** **Patient and donor characteristics**.

Variable			n (%) or mean (SEM)
Recipient characteristics			
	Age, years		34.9 ± 0.4
	Race		
		White	241 (36)
		Black	345 (51)
		Other	91 (13)
	BMI, kg/m^2^		27.2 ± 0.2
	T2D		589 (87)
	CVD		32 (5)
	Smoking		155 (23)
	Malignancy		27 (4)
	ICD		371 (55)
	Dialysis		15 (2)
	IVDU		95 (14)
	Days on Status 1		0.4 ± 0.2
	Days on Status 2		2.4 ± 0.3
	Days on Status 3		5.2 ± 1.9
	Days on Status 4		19.4 ± 3.8
	Days on Status 1A		20.4 ± 1.7
	Days on Status 1B		60.3 ± 5.8
	LVAD at listing		72 (11)
	RVAD at listing		10 (2)
	TAH at listing		68 (11)
	On ventilator at listing		26 (4)
	Inotropes at listing		287 (42)
	Prior cardiac surgery		135 (20)
	Creatinine, mg/dL		1.08 ± 0.02
	CPRA value		29.2 ± 2.3
	Cardiac output, L/min		4.1 ± 0.1
	PCWP, mmHg		20.1 ± 0.4
	mPAP, mmHg		27.9 ± 0.4
	IABP at listing		67 (10)
	IABP at Tx		95 (14)
	ECMO at listing		13 (2)
	ECMO at Tx		20 (3)
Donor characteristics			
	Age, years		30.5 ± 0.4
	Race		
		White	419 (62)
		Black	123 (18)
		Other	135 (20)
	Female gender		325 (48)
	BMI		26.2 ± 0.2
	Creatinine, mg/dL		1.45 ± 0.05
	T2D		20 (3)
	LVEF, %		61 ± 0.3
	Ischemic time, hours		3.3 ± 0.1
	Distance (miles)		222 ± 9

SEM, standard error of the mean; BMI, body mass index; T2D, type 2 diabetes; CVD, cardiovascular disease; ICD, implantable cardiac defibrillator; IVDU, intravenous drug user; LVAD, left ventricular assist device; RVAD, right ventricular assist device; TAH, total artificial heart; CPRA, calculated panel reactive antibody; PCWP, pulmonary capillary wedge pressure; mPAP, mean pulmonary arterial pressure; IABP, intra-aortic balloon pump; Tx, transplant; ECMO, extracorporeal membrane oxygenator; LVEF, left ventricular injection fraction.

Regarding donor characteristics, the mean donor age was 30.5 ± 0.4 years, and most donors were white (n = 419, 62%). Almost half were female (n = 325; 48%) with a mean creatinine and LVEF of 1.45 ± 0.05 mg/dL and 61 ± 0.3%, respectively. The mean ischemic time was 3.3 ± 0.1 hours, and the average distance from donor to recipient was 222 ± 9 miles (Table 1).

Older age was associated with lower overall mortality (hazard ratio (HR): 0.97; 95% CI: 0.95, 0.98; *p* < 0.01), while diabetes (HR: 1.01; 95% CI: 1.01, 1.01; *p* < 0.01), dialysis (HR: 1.01; 95% CI: 1.01, 1.01; *p* < 0.01), days on Status 1 (HR: 1.06; 95% CI: 1.03, 10.9; *p* < 0.01), creatinine (HR: 1.29; 95% CI: 1.02, 1.64; *p* = 0.034), and length of stay (HR: 1.01; 95% CI: 1.01, 1.02; *p* = 0.02) were associated with a higher risk of overall mortality. The 30-day mortality was 2.8%, and 1-year mortality was 11.1% (Fig. 1B). Unadjusted Cox regression analysis revealed CPRA and Status 1 (old allocation) as new risk factors for 30-day mortality (odds ratio (OR): 1.03; 95% CI: 1.01, 1.05; *p* < 0.01; OR: 1.06; 95% CI: 1.01, 1.13; *p* < 0.01, respectively). No risk factor was identified for 1-year mortality. Extracorporeal membrane oxygenation (ECMO) was a risk factor for stroke postoperatively (OR: 7.9; 95% CI: 1.6, 37.9; *p* < 0.05). Higher age and creatinine levels pretransplant were associated with higher risk for dialysis (OR: 1.03; 95% CI: 1.02, 1.05; *p* < 0.01; OR: 1.66; 95% CI: 1.07, 2.72; *p* < 0.01, respectively). Increased pulmonary capillary wedge pressure (PCWP) was associated with a lower need for permanent pacemaker implantation (OR: 0.90; 95% CI: 0.81, 0.99; *p* < 0.01), and increased age was associated with a lower incidence of rejection requiring treatment (OR: 0.97; 95% CI: 0.95, 0.98; *p* < 0.01). Era effect was prominent in the case of 1-year mortality (OR: 0.95; 95% CI: 0.91, 0.99, *p* = 0.006) and marginal in the case of 30-day mortality (OR: 0.93; 95% CI: 0.87, 1.00; *p* = 0.052).

The most common postoperative complication was renal failure requiring dialysis (n = 59; 9%), followed by stroke (n = 17; 3%) and permanent pacemaker implantation (n = 8; 1%). A total of 202 patients (30%) required treatment for graft rejection within one year of the transplant. The mean length of hospital stay was 19.9 ± 0.6 days.

No significant differences in mortality or secondary outcomes were observed when comparing the old with the new allocation systems.

When comparing females who underwent HTx for postpartum cardiomyopathy to all the other females in the UNOS databases who underwent HTx for different reasons, an increased association with postoperative mortality (HR: 1.42; 95% CI: 1.25, 1.61; *p* < 0.001) and rejection requiring treatment within one year (OR: 1.93; 95% CI: 1.61, 2.32; *p* < 0.001) was observed.

## 4. Discussion

This study offers a comprehensive insight into the outcomes of patients with postpartum cardiomyopathy after HTx based on nationwide data from the USA over the past two decades. The average age of the recipients was 35 years, and half were black, while nine out of ten had T2D. Younger age, diabetes, pretransplant dialysis, days on Status 1, and creatinine were associated with higher mortality, while an era effect was observed for 1-year mortality.

A population-based study from the late 90s utilizing the National Hospital Discharge Survey showed that the incidence of postpartum cardiomyopathy is approximately 0.03% and is associated with 1.4% and 2% inpatient and one-year mortality, respectively [2]. A prospective multicenter study (Investigations of Pregnancy Associated Cardiomyopathy; IPAC study) of 100 women with postpartum cardiomyopathy showed that 3% had experienced major events or had persistent severe cardiomyopathy (ejection fraction <35%) within the first year [3]. A European study of 66 patients showed that 5% of the patients did not recover from postpartum cardiomyopathy, and 2% had died at a 5-year follow-up [4]. A retrospective analysis of the Interagency Registry for Mechanically Assisted Circulatory Support showed that the 2-year mortality of women with postpartum cardiomyopathy who underwent left ventricular assist device implantation was 17%. A single-center study of 14 women with postpartum cardiomyopathy who underwent heart transplants reported a 7% 1-year mortality and an overall mortality of 21.5% during a median follow-up of 7.7 years [4]. A comparative study of women undergoing heart transplants for postpartum cardiomyopathy versus other etiologies showed comparable outcomes between the two groups [4].

We also observed an increased risk for mortality and rejection associated with postpartum cardiomyopathy as the etiology of heart failure requiring HTx, which agrees with a previous UNOS study [5]. The observed era effect can be attributed to better peripartum management of these patients and optimized general medical management of heart failure.

Interpretation and extrapolation of our study’s results should be performed cautiously, given certain limitations associated with its design. This is a retrospective cohort utilizing the UNOS database; thus, these data provide a snapshot of the reported evidence over the past two decades and do not include information derived from a prospective randomized study designed to address our primary and secondary objectives. Furthermore, these are nationwide derived data specific only to the USA population and may not apply to other populations.

## 5. Conclusions

In conclusion, our results show that younger age, diabetes, pretransplant dialysis, days on Status 1, and creatinine are associated with higher mortality, while an era effect was observed for 1-year mortality in patients with postpartum cardiomyopathy after HTx. Further studies are required to investigate the role of heart transplants in postpartum cardiomyopathy refractory to medical management.

## Availability of Data and Materials

The datasets used and/or analyzed during the current study are available from the corresponding author on reasonable request.
